# Compression therapy following posterior lumbar interbody fusion: a prospective, randomized, clinical study

**DOI:** 10.1186/s12893-019-0612-7

**Published:** 2019-11-05

**Authors:** Wu Sun, Jing-hua Gao, Li-guo Zhu, Wei Xiao, Zhen-zhong Wang, Ke-xin Yang, Qing Zhang, Bao-jian Wang

**Affiliations:** 1 Spine Department 2, Wangjing Hospital, China Academy of Chinese Medical Sciences, Beijing, 100102 People’s Republic of China; 2 State Key Laboratory of Pharmaceutical New-Tech for Chinese Medicine, Jiangsu Kanion Pharmaceutical Co. Ltd., Lianyungang, 222001 People’s Republic of China

**Keywords:** Lumbar spine surgery, Compression therapy, Pain, Anemia

## Abstract

**Background:**

Wound-related complications are an inevitable issue faced by spinal surgeons. Negative pressure drainage remains the most commonly used method to prevent postoperative hematoma and related complications. This prospective, randomized, controlled study was conducted to evaluate the efficacy of compression therapy following posterior lumbar interbody fusion, with emphasis on pain, anemia, and inflammation.

**Methods:**

Sixty consecutive patients who have undergone posterior lumbar interbody fusion in the age range 43–78 years, with an average age of 59 years, were selected and randomly assigned into two groups. Factors, such as drainage volume, visual analog scale (VAS) pain score for back pain, white blood cell (WBC) count, red blood cell (RBC) count, hemoglobin (Hb) levels, erythrocyte sedimentation rate (ESR), and C-reactive protein (CRP) levels assessed on the 1st, 3rd, and 10th days postoperatively, were compared between the two groups.

**Results:**

The average follow-up was 6 months, ranging from 3 to 11 months. Drainage volume, VAS score, and CRP levels on the 10th day after the surgery were found to be significantly lower in the treatment group than in the control group. RBC count and Hb levels on the 3rd and 10th postoperative days were observed to be significantly higher in the treatment group than in the control group (*P* < 0.05). During discharge, the wounds of the patients of the both groups had healed and neither showed any symptoms of infection, hematoma, or necrosis.

**Conclusion:**

Compression therapy relieves pain, alleviates anemia, and the inflammatory response following posterior lumbar interbody fusion.

**Trial registration:**

ChiCTR1800015825 on chictr.org.cn, April 23, 2018, the trial registry is Chinese Clinical Trial Registry.

## Background

As the older population continues to increase worldwide, degenerative lumbar disease is becoming a growing cause of concern among healthcare providers [1, 2]. For patients with no effect of conservative treatment, posterior decompression is the most common treatment [3, 4]. However, to get visual field exposure, the paravertebral muscle has to be separated on a large scale and stretched for an extended period of time during the posterior operation. Soft tissue edema and hematoma increase the tension on incisions, cause pain and fever, and even lead to wound infections in the operative field [5, 6]. Moreover, epidural hematoma may result in spinal cord compression and even cause paralysis [7]. The above-mentioned complications not only impair clinical outcomes among patients with internal fixation but also notably increase the risk of implant infection [8].

Currently, negative pressure drainage is the most popular method for preventing postoperative hematoma and related complications [9, 10]. Negative pressure drainage has shown to accelerate wound healing by advancing angiogenesis, improving microvascular blood flow, triggering granulation tissue formation, and decreasing edema [11]. Contrarily, a few studies have shown that negative pressure drainage is not beneficial in spinal surgery [12, 13]. Closed suction drainage is another method that could intensify postoperative blood loss and require transfusion [14]. Thus, utilizing negative pressure drainage method in posterior spinal surgery is controversial.

Compression therapy helps in reducing the pain, and the blood flow prevents the development of edema, swelling, and hemarthrosis, protects soft tissues [15], increases the range of motion, and improves functioning [16]. Therefore, it has been widely used to avert deep venous thrombosis [17], edema management, ankle fracture [18], shoulder/knee arthroscopy, and wound care [15–17]. However, no reports were documented on the usage of compression therapy post posterior lumbar interbody fusion.

Therefore, this prospective, randomized, clinical study aimed to investigate the effect of compression therapy on the postoperative course of posterior lumbar interbody fusion, especially on pain, anemia, and inflammatory reactions.

## Methods

Consolidated Standards of Reporting Trials (CONSORT statement) was followed in this randomized unblinded prospective study, reviewed and approved by the Medical Ethics Committee of Wangjing Hospital of the China Academy of Chinese Medical Sciences (WJEC-KT-2017-013-P002), and was preregistered in the Chinese Clinical Trial Registry (ChiCTR1800015825). All records of the patients involved were kept confidential.

The use of negative pressure drainage method is controversial, and literature reports on compression therapy for the postoperative course of posterior lumbar interbody fusion are lacking. Therefore, 60 patients were recruited for investigating the effect of compression therapy on the postoperative course of posterior lumbar interbody fusion; these patients were randomly allocated to two groups.

A total of 76 patients with lumbar spinal stenosis, which was diagnosed through clinical symptoms, computed tomography, and magnetic resonance imaging (MRI) results underwent posterior lumbar decompression, internal fixation, and interbody fusion performed by the first author during the period May 2018 to January 2019. Of them, 60 patients were enrolled for this study. The treatment group patients were operated on segments including 2 cases in L3/4; 9 each in L4/5 andL5/S1; 2 in L3–5; 1in L3-S1; and 7 in L4-S1, whereas the control group included 4 cases in L4/5; 12 in L3–5; 4 in L3-S1; and 12 in L4-S1. All patients provided written informed consent of their treatment data and related pictures for public use. The inclusion and exclusion criteria are listed in Table 1.

**Table 1:** Inclusion and exclusion criteria

Inclusion criteria	Exclusion criteria
Age > 40 years [19]	≤40 years of age
Low back pain/intermittent claudication	Decompression without internal fixation [8]
CT/MRI confirmed lumbar spinal stenosis	Back or leg pain of unknown etiology
Failed conservative treatment for 2 weeks	Systemic or local infections
Posterior lumbar decompression, internal fixation, and interbody fusion	Chronic steroid use, diabetes, hemato-oncological disease, renal disease, autoimmune disease [20]
	Blood Transfusion in perioperative period

Patients ≤40 years of age or with other risk factors [8, 19, 20] were excluded from the study. Only patients aged > 40 years exhibiting typical clinical symptoms of lumbar spinal stenosis, and radiological confirmation were included.

A random number generator was used to assign patients either to the treatment group (closed suction drain [CSD] with compression therapy) or the control group (CSD alone, also called negative wound pressure therapy). Standard surgical procedure was followed for all patients.

**Fig. 1:**
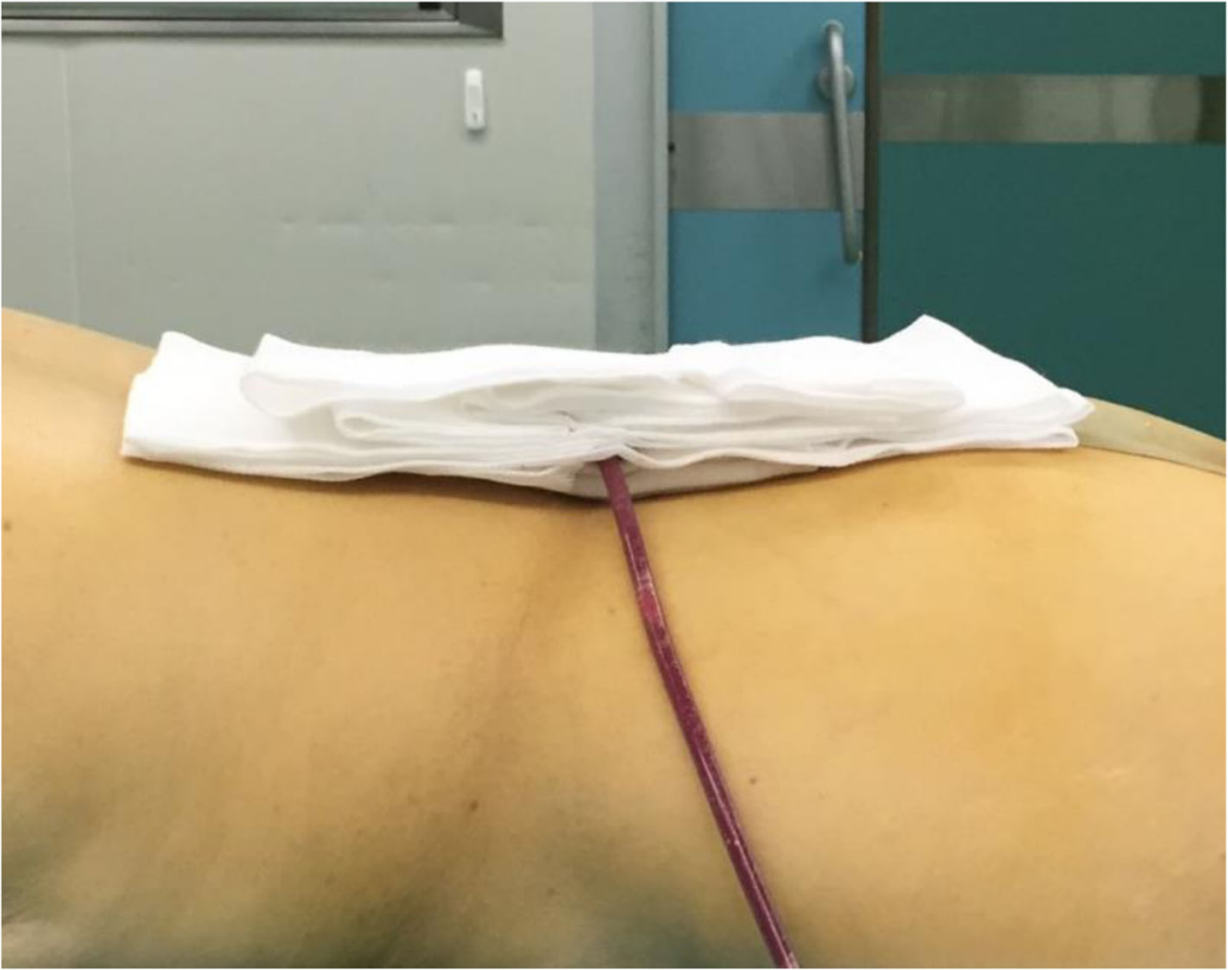
Dressing was folded into a shuttle shape to counteract the lumbar lordosis

**Fig. 2:**
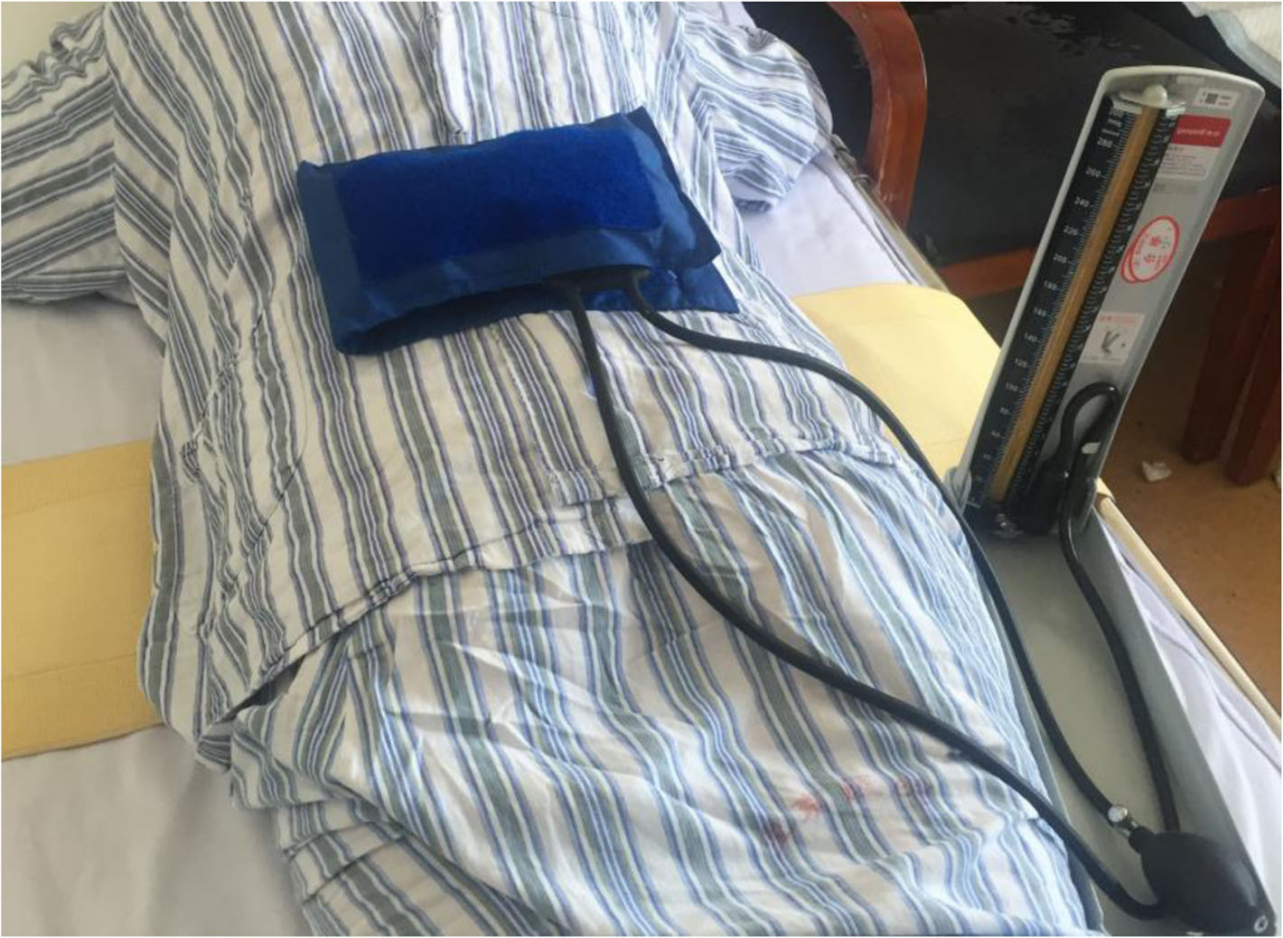
Cuff sphygmomanometer used for marking lines at 20 and 40 mmHg

**Fig. 3:**
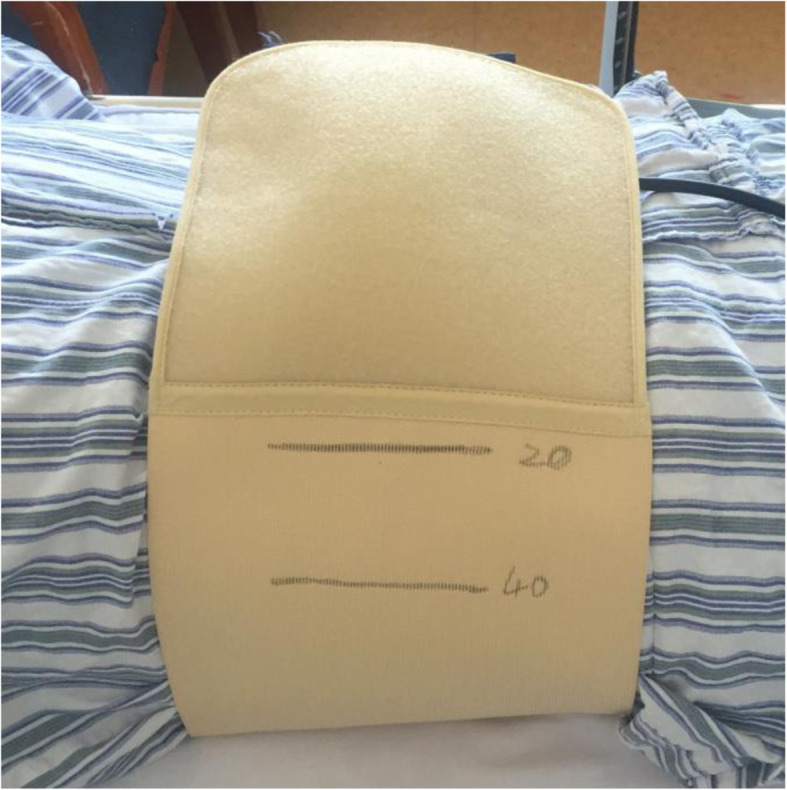
Cuff sphygmomanometer used for marking lines at 20 and 40 mmHg

**Fig. 4:**
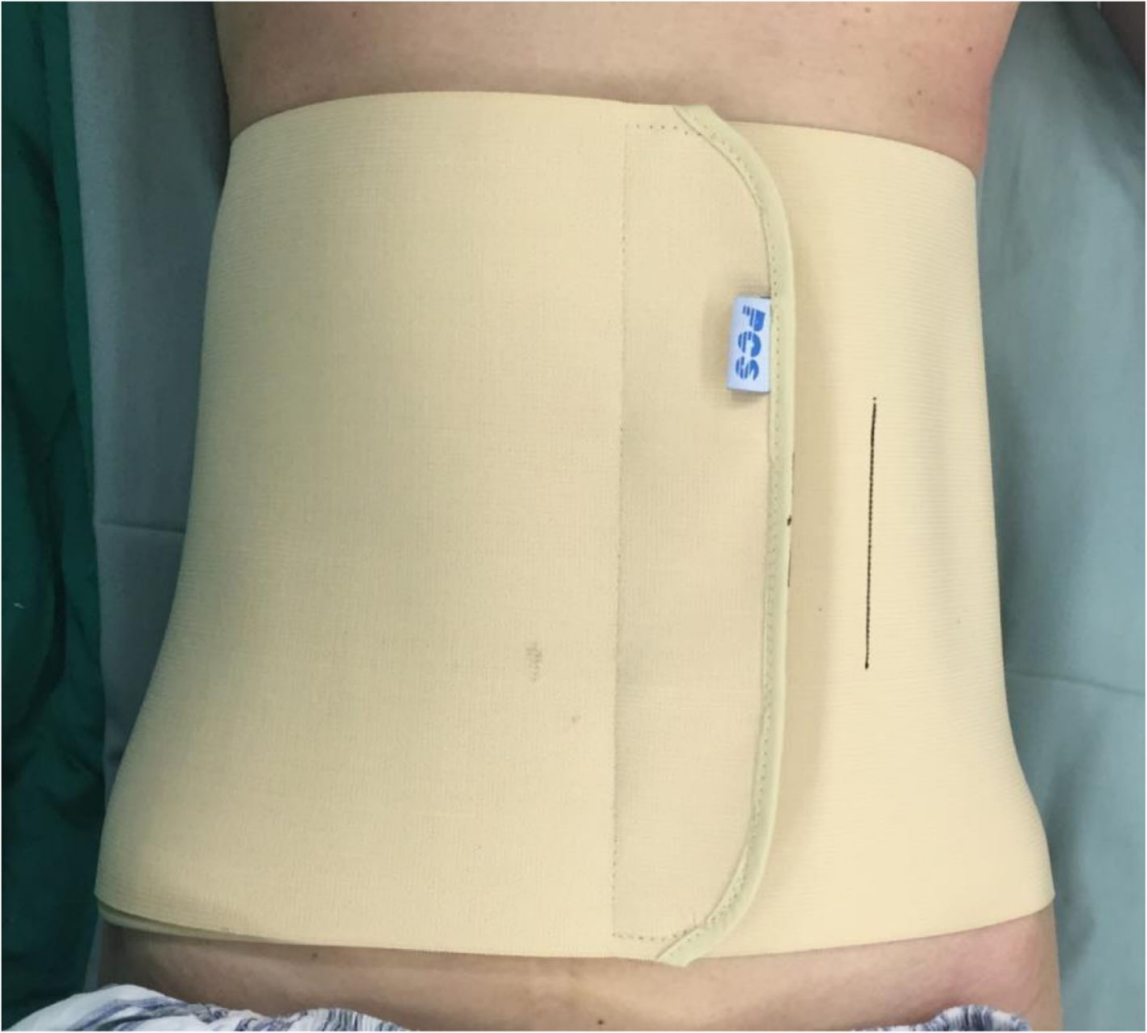
Elastic waist band worn between the two marked lines

For patients of the treatment group (CSD with compression therapy), a 16 Fr silicone CSD (Fr-16; Shandong Branden Medical Devices Co., Ltd., Shandong City, China) was inserted into the surgical area. All CSDs were used with mild suction pressure (half negative). A sterile gauze bandage was used, and the aseptic dressing was folded into a shuttle shape (Fig. 1) for compression therapy postoperatively, which was preferably thick to counteract the lumbar lordosis. Simultaneously, an elastic waist band (PCS-5011; Rehan Health Care Co., Ltd., Shanghai City, China) was used for pressure. The cuff of the cuff sphygmomanometer was laid in the middle of the patients’ waist in prone position, the inflatable valve tightened when the mercury column was about to rise, pressed with an elastic waist band, and marked when the pressure reached 20 mmHg and 40 mmHg [21], respectively (Figs. 2 and 3). The elastic waist band was worn at the 40 mmHg marking lines as far as possible post operation. If the patients were found with breathing difficulty or abdominal discomfort, the elastic waist band was loosened as per the patient’s preference, but not exceeding the 20 mmHg marking line (Fig. 4). If relaxing the elastic waist band up to the 20 mmHg still did not relieve the above mentioned symptoms, the elastic waist band was removed and the patient was dropped out of the study. Usually, the first dressing was changed when the drainage was removed. During this, the patient was laid in the prone position, and a sterile dressing was folded into a shuttle shape and then pressurized and bandaged. The dressing was changed in case of abnormal conditions such as increased wound pain or blood oozing. Otherwise, it was changed for the second time when the stitches were removed, and each time a dressing was changed, a new sterile dressing was used, followed by compression dressing. The elastic waist band was worn until the wound healed completely post operation. In the control group, as described in the similarly treatment group, CSD alone without the wound pressure therapy was administered.

The patient’s back pain was assessed with the visual analog scale (VAS). The VAS score indicating the most painful back wound post operation was recorded. When the amount of bleeding did not exceed 100 mL per day, the patients’ total drainage volume was recorded postoperatively by removing the CSD. White blood cell (WBC) count, red blood cell (RBC) count, hemoglobin (Hb) levels, erythrocyte sedimentation rate (ESR), and C-reactive protein (CRP) levels [22] on the 1st, 3rd, and 10th days postoperatively were recorded. These indicators were compared between the two groups to evaluate the effect of compression therapy on postoperative posterior lumbar interbody fusion. All 60 patients were available for follow-up, during which postoperative complications were recorded. The mean duration of the follow-up was 6.25 ± 2.36 months.

Statistical analysis was performed using SPSS 22.0 software (IBM Corp., Armonk, NY, USA). The data measured are presented as the mean values (standard deviation). The Kolmogorov–Smirnov test and histograms indicated a nonparametric distribution of the data. Differences of outcome measures between the two groups and their significant values were analyzed using the independent sample test or Mann–Whitney U test (*p* < 0.05) depending on normal distribution. Chi-square test was used to compare the numeration data between the two groups.

## Results

**Fig. 5:**
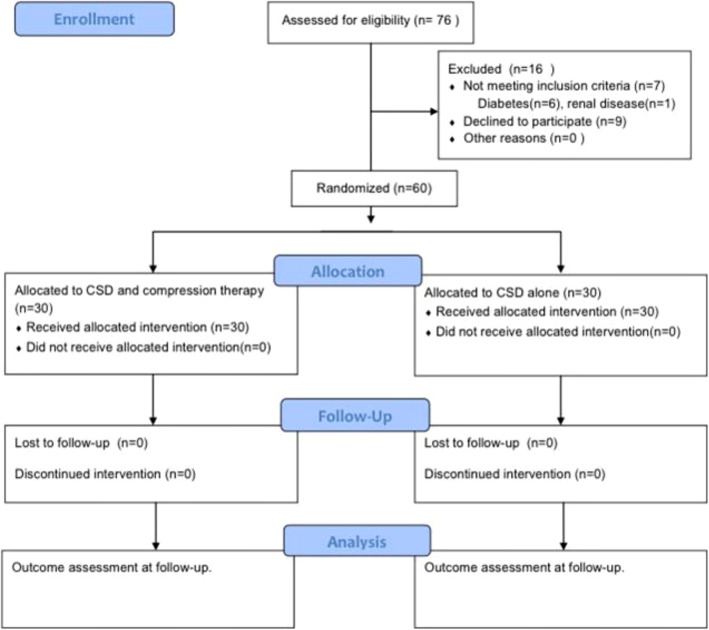
CONSORT flowchart exhibiting the recruitment process of participants into the trial

A CONSORT flowchart is presented in Fig. 5 demonstrating the recruitment, allocation, and flow of the trial. The number of patients treated under the two groups, namely, CSD along with compression therapy and CSD alone, was 30 in each group. Table 2 exhibits the comparison of patients’ demographics and known risk factors for wound healing between the two groups prior to the intervention. No significant differences in these data were observed.

**Table 2:** Comparison of general data and laboratory indices between the treatment and control groups before the intervention

	Treatment group (*n* = 30)		Control group (*n* = 30)					
	n	Mean (SD)	n	Mean (SD)	χ^2^-value	z-value	t-value	*p*-value
Age		57.77 (9.02)		60.43 (9.06)	–	–	−1.14	0.26
Gender					0.07	–	–	0.79
Male	12		11					
Female	18		19					
BMI		26.11 (3.61)		26.51 (3.06)	–	–	−0.46	0.64
Surgical segment (number)		2.40 (0.56)		3.00 (0.52)	–	−3.89	–	0.09
Surgery duration (min)		175.67 (42.48)		180.67 (30.05)	–	–	−0.53	0.60
Blood loss (mL)		282.33 (92.87)		337.33 (131.20)	–	−1.58	–	0.11
Laboratory indexes								
WBC (10^9/L)		5.97 (1.55)		5.91 (1.42)	–	–	0.16	0.87
RBC (10^9/L)		4.63 (0.46)		4.54 (0.49)	–	–	0.73	0.47
Hb (g/L)		140.33 (15.40)		140.30 (14.42)	–	–	0.01	0.99

In the treatment group, 5 patients complained breathing problems and 9 patients abdominal discomfort when the elastic waist band was worn near the 40 mmHg marking line. The elastic waist band was loosened to the 20 mmHg marking line to minimize these symptoms, and no patient was dropped out of the study. Values of the drainage volume, VAS scores, and CRP levels on the 10th day postoperation were found to be significantly lower in the treatment group compared with the control group. The RBC count and Hb levels recorded on the 3rd and 10th days after the operation were significantly higher in the treatment group compared with the control group (all *p* < 0.05, Table 3). When being discharged, the wounds of the patients of the both groups had healed and neither showed any symptoms of infection, hematoma, or necrosis, and no signs of late infection were found in either group during the follow-up.

**Table 3:** Comparison of general data and laboratory indices between the treatment and control groups after the intervention

	Treatment group (*n* = 30)	Control group (*n* = 30)			
	Mean (SD)	Mean (SD)	z-value	t-value	*p*-value
Drainage volume (mL)	332.33 (131.98)	447.00 (178.46)	–	−2.83	0.006*
Maximum VAS (cm)	3.07 (1.20)	4.00 (1.13)	−3.26	–	0.001*
WBC^1^ (10^9/L)	12.78 (3.21)	12.60 (2.99)	–	0.223	0.825
RBC^1^ (10^9/L)	4.02 (0.70)	3.76 (0.49)	–	1.656	0.103
Hb^1^ (g/L)	119.10 (15.17)	116.1 (14.38)	–	0.786	0.435
ESR^1^ (mm/h)	13.97 (8.59)	12.80 (10.67)	–	0.466	0.643
CRP^1^ (mg/L)	24.20 (16.81)	32.35 (18.77)	–	−1.771	0.082
WBC^3^ (10^9/L)	10.83 (2.76)	10.20 (2.27)	–	0.964	0.339
RBC^3^ (10^9/L)	3.84 (0.50)	3.46 (0.53)	–	2.896	0.005*
Hb^3^ (g/L)	116.77 (13.54)	107.1 (15.99)	–	2.527	0.014*
ESR^3^ (mm/h)	32.30 (22.65)	34.73 (26.96)	–	−0.378	0.706
CRP^3^ (mg/L)	44.06 (48.64)	60.23 (51.43)	–	−1.251	0.216
WBC^10^ (10^9/L)	8.48 (1.77)	7.57 (2.38)	–	1.676	0.099
RBC^10^ (10^9/L)	5.68 (2.27)	4.66 (1.54)	–	2.040	0.047*
Hb^10^ (g/L)	120.37 (14.77)	109.53 (22.26)	–	2.221	0.030*
ESR^10^ (mm/h)	34.90 (20.01)	34.59 (22.31)	–	0.056	0.955
CRP^10^ (10^9/L)	13.87 (11.24)	23.14 (19.30)	–	−2.273	0.028*

*Statistical difference (*p* < 0.05)

^1^First day after the operation; ^3^3rd day after the operation; ^10^10th day after the operation

The postoperative drainage volume and VAS scores in the treatment group were found to be significantly lower than those of the control group. These findings suggest that compression therapy reduced drainage besides relieving pain indeed.

**Fig. 6:**
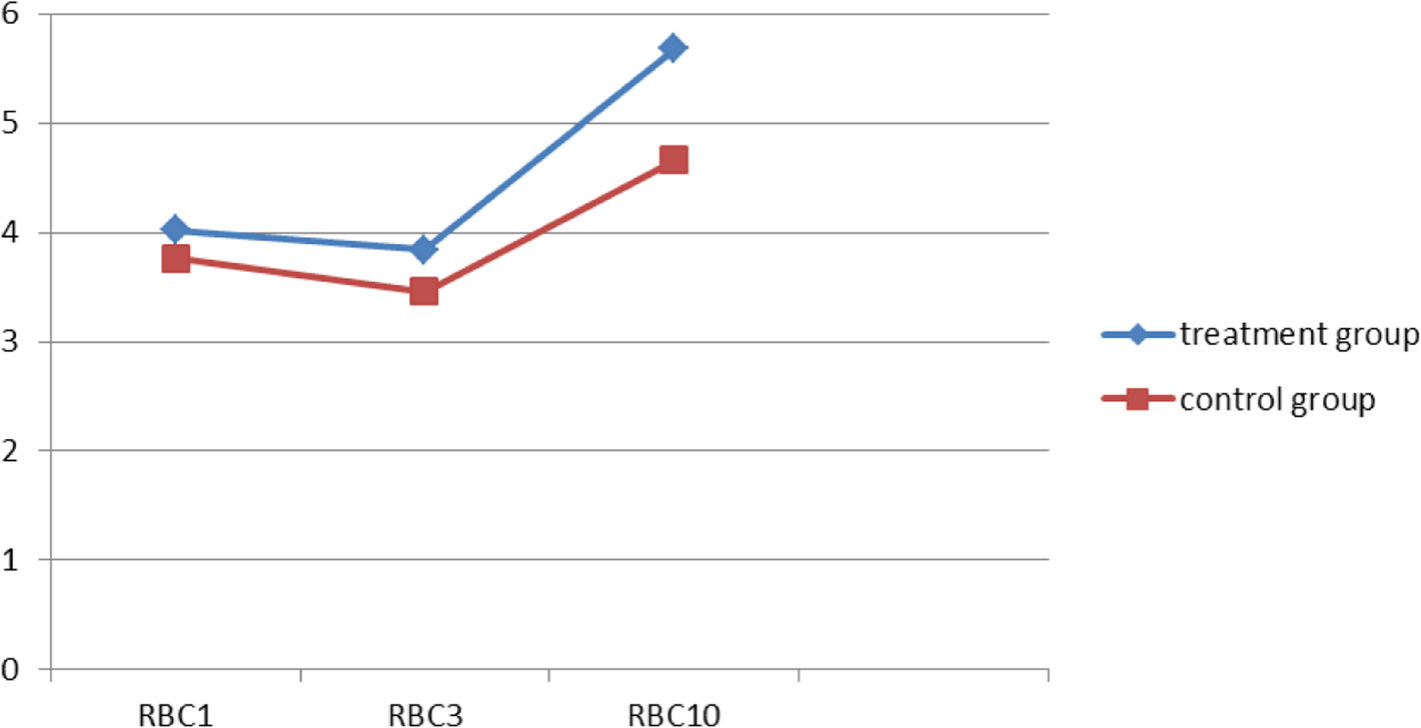
Comparative graphical representation of changes in the RBC count (10^9/L) in the treatment and control groups

**Fig. 7:**
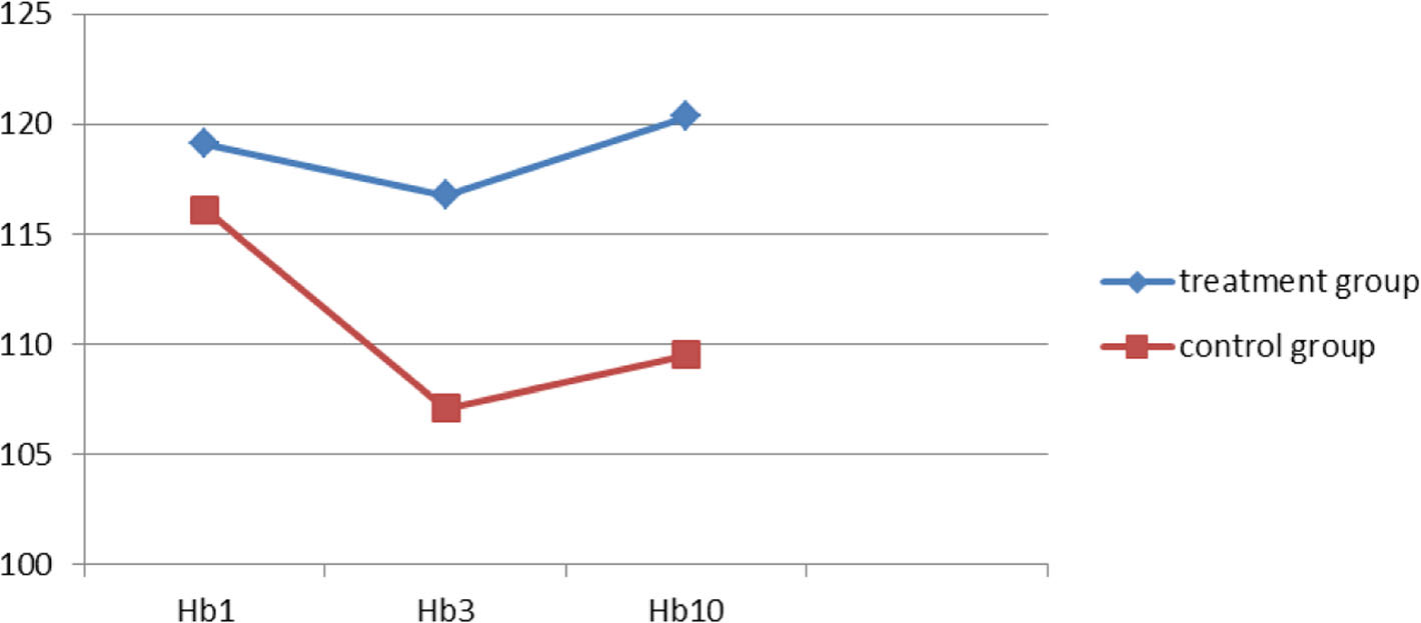
Comparative graphical representation of changes in Hb levels (g/L) in the treatment and control groups

**Fig. 8:**
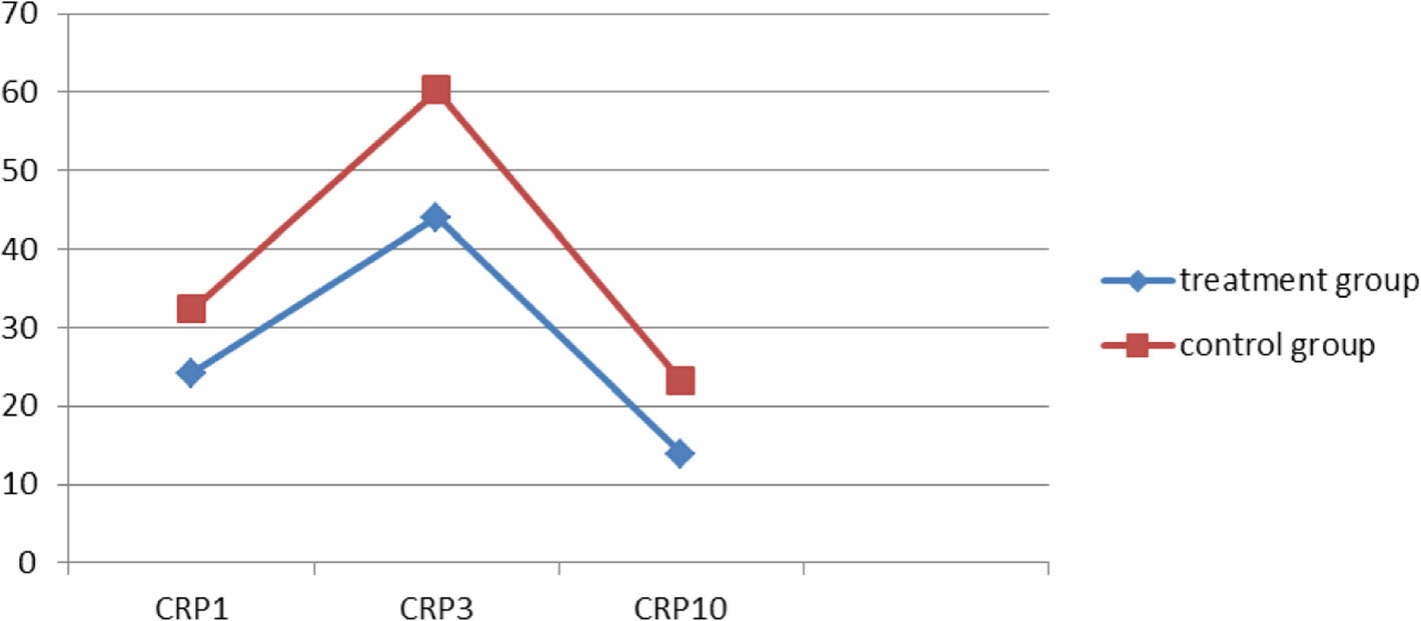
Comparative plots of changes in CRP levels (mg/L) in the treatment and control groups

The RBC count and Hb levels on the 3rd and 10th days post operation were recorded to be significantly higher in the treatment group compared with those in the control group. However, CRP levels on the 10th day post operation were observed to be significantly lower in the treatment group than those in the control group (Figs. 6, 7 and 8). These findings suggest that compression therapy improved the condition of anemia and the inflammatory response.

## Discussion

The posterior approach is the most commonly used technique for lumbar spinal surgery due to the safety of the approach and clarity of exposure. Negative pressure drainage has been the most popular technique used in preventing postoperative hematoma and related complications [9–11]. However, the range of incidence of wound-related complications after spinal surgery was 0.4–20% [23–25], which are an inevitable problem faced by spinal surgeons. Thus, the usage of negative pressure drainage in posterior spinal surgery is controversial [12–14].

Compression therapy is widely used for treating complex wound healing and scar hyperplasia [15–18], but there are no reports on this therapy being utilized after posterior lumbar surgery. Clinically, the usage of an elastic waist band post operation is known to relieve pain of the surgical wound while turning over. Sixty patients were selected to assess the effect of compression therapy on posterior lumbar interbody fusion. For the convenience of comparability, factors affecting the incidence of wound complications post lumbar surgery, such as chronic steroid use and diabetes, were excluded [8, 19, 20]. To offset lumbar lordosis, the aseptic dressing was folded into a shuttle shape. Compression therapy was detected to relieve symptoms such as fever, anemia, and the inflammatory response following posterior lumbar interbody fusion.

The mechanisms of how compression therapy relieves these effects are as follows: (1) Mechanical stress produced by the elastic waist band causes paravertebral muscles around the incision to move toward the center, which reduce the dead space thus reducing the incidence of hematomas in the operative cavity [26]. (2) The aseptic dressing folded into a shuttle shape offset lumbar lordosis, which decreased the negative pressure in the surgical cavity, further reducing exudation and release of inflammatory mediators. (3) Mechanical stress tends to minimize the partial pressure of oxygen in the wound tissue and stimulate the initiation of repair. This condition is useful to timely elimination of necrotic tissue and a reduction in the release of inflammatory mediators in the wound [26]. (4) Mechanical stress controls the collagen synthesis by limiting the blood and oxygen supply, thereby reducing collagen production and encouraging realignment of existing collagen bundles thus accelerating healing of the wound [27].

The major concern regarding the routine use of an elastic waist band is the additional expenditure incurred in the procedure. Usually, the equipment cost per patient is $20, and no additional expenses are incurred for using the elastic waist band. Moreover, coordination of dressing changes with a special nurse or wound specialist is not necessary. Therefore, compression therapy is useful for primary medical organizations.

Some of the limitations of our study are discussed as follows. (1) If elasticity of the elastic band decreases during its use, sustaining a fixed pressure on the surgical wound turns difficult. (2) No postoperative MRI examination for evaluating postoperative hematomas in the operative area is performed. (3) Owing to the small sample size and the single-center design, the study findings are limited. To further confirm our findings, an immediate attention is required for the multicenter, randomized, controlled study.

## Conclusion

This is the first study to demonstrate the effect of compression therapy for treating postoperative posterior lumbar interbody fusion. Positive factors, such as the curative effect, low cost, simple operation, and high compliance of patients, indicate using compression therapy on postoperative posterior lumbar interbody fusion should be considered as part of the postoperative therapy for enhancing recovery.

## Data Availability

Data sets analyzed during the current study are available with the corresponding author and can be obtained on reasonable request.

## Abbreviations

CRP: C-reactive protein
CSD: Closed suction drain
ESR: Erythrocyte sedimentation rate
Hb: Hemoglobin
MRI: Magnetic resonance imaging
RBC: Red blood cell
VAS: Visual analog scale
WBC: White blood cell
